# Dynamical Analysis of Hyper-ILSR Rumor Propagation Model with Saturation Incidence Rate

**DOI:** 10.3390/e25050805

**Published:** 2023-05-16

**Authors:** Xuehui Mei, Ziyu Zhang, Haijun Jiang

**Affiliations:** College of Mathematics and System Science, Xinjiang University, Urumqi 830046, China; meixuehui163@163.com (X.M.); zhang_zy1111@163.com (Z.Z.)

**Keywords:** hyper-ILSR, hypergraph, saturation incidence rate

## Abstract

With the development of the Internet, it is more convenient for people to obtain information, which also facilitates the spread of rumors. It is imperative to study the mechanisms of rumor transmission to control the spread of rumors. The process of rumor propagation is often affected by the interaction of multiple nodes. To reflect higher-order interactions in rumor-spreading, hypergraph theories are introduced in a Hyper-ILSR (Hyper-Ignorant–Lurker–Spreader–Recover) rumor-spreading model with saturation incidence rate in this study. Firstly, the definition of hypergraph and hyperdegree is introduced to explain the construction of the model. Secondly, the existence of the threshold and equilibrium of the Hyper-ILSR model is revealed by discussing the model, which is used to judge the final state of rumor propagation. Next, the stability of equilibrium is studied by Lyapunov functions. Moreover, optimal control is put forward to suppress rumor propagation. Finally, the differences between the Hyper-ILSR model and the general ILSR model are shown in numerical simulations.

## 1. Introduction

Rumors are inaccurate information, which may be subjective assumptions based on partial facts. With the popularity of social media platforms, rumors propagate faster and more widely, endangering the social stability and affecting people’s lives [1]. For example, on the 8 September 2022, a netizen spread a rumor on WeChat that said “The Wuzhou city in Guangxi Province will be on lockdown at midnight!”, causing public panic and disrupting the order of epidemic prevention and control. Thus, it is important to investigate the propagation mechanism of rumors to effectively reduce the harm caused by rumor transmission.

The study of rumor spreading began decades ago, based on the similarities to the spread of diseases. Daley and Kendall [2] created the DK rumor transmission model in 1964, which was modified by Maki and Thompson [3] in 1973; however, they ignored the impact of the network topology on the spread of rumors. In 2001, a rumor-spreading model in small-world networks was first constructed by Zanette [4]. Then, many scholars considered rumor propagation models in complex networks [5,6,7,8,9]. Moreover, because of the different degrees of nodes, the transmission dynamics of rumors in heterogeneous networks were discussed in different models, such as the I2SR (ignorants–spreaders1–spreaders2–removers) model [10], I2S2R (ignorants–spreaders1–spreaders2–stiflers1–stiflers2) rumor model [11], IE2S2R (ignorants–exposures–spreaders1–spreaders2–stiflers1–stiflers2) model [12], and SHILR (susceptible–hesitating–infected–latent–recovered) model [13]. These models presumed that the incidence was to be proportional to the number of spreaders, which was unrealistic. Because a person’s connection with others is limited, the contact rate tends to be saturated when the number of spreaders is large enough. Capasso and Serio [14] put forward a saturation incidence rate $g\left(I\right)S=\frac{kIS}{1+\alpha I}$ to replace bilinear incidence, in which $g\left(I\right)=\frac{kI}{1+\alpha I}$ and α>0, *I* and *S* denotes infective and susceptible individuals, respectively, *k* is the infection rate and α is the psychological influence factor. Then, on this basis, epidemic models with saturation incidence were established in [15,16,17,18]. Chen [19] proposed an SEIR (susceptible–exposed–infected–recovered) rumor-spreading model with saturated incidence in heterogeneous networks. An ISCR (ignorants–spreaders–cooled–removers) model with saturated incidence was investigated by Yue and Huo [20] on a scale-free network. Nevertheless, the above models ignore the higher-order interactions of rumor dissemination. In the rumor propagation process, people receive rumors from individuals or groups. Furthermore, individuals are more likely to believe the rumor if they are exposed to different spreaders in the same group.

To present higher-order interactions, scholars adopted different tools [21,22,23]. Schaub and Benson [24] used the Hodge Laplacian to analyze higher-order interactions. Simplicial complexes were introduced by Iacopini to study higher-order interactions in social contagion [25]. Additionally, some scholars proposed the stability conditions of different order interactions [26,27]. Moreover, Arruda and Petri [28] analyzed the dynamics of the social contagion model on hypergraphs. In [29], some researchers discussed the effect of group interactions on collaboration. Restrepo and Landry [30] studied the impact of heterogeneity on the hypergraph spreading model. A Hyper-SIR rumor propagation model was given by Zhang and Mei [31] based on hypergraph theory. Compared with other tools, hypergraphs can directly reflect the higher-order interactions between different individuals, which can describe the topology of the social and communication networks better.

It is worth noting that few scholars have studied the rumor propagation model with saturation incidence described by the hypergraph. The traditional ILSR (ignorant–lurker–spreader–recover) model is constructed based on a simple graph, with edges representing the connection between two nodes. This model only considers the “point-to-point” spread of rumors, but ignores the “point-to-group” spread. Additionally, because of the bandwagon effect, the probability that people believe a rumor is not simply proportional to the number of times they hear the rumor, but there is an additional infection rate. Enlightened by the discussion above, based on hypergraph theories, a new Hyper-ILSR rumor model in heterogeneous networks is constructed. The main improvements are as follows:

(1) To represent the higher-order interactions in the process of rumor-spreading, hypergraph theories are applied in the model. Individuals do not believe the rumor when they first hear it, but may believe it when they hear it from multiple individuals—this is the higher-order interaction.
(2) To formulate a more reasonable rumor model, saturation incidence is used in the Hyper-ILSR model. Most models take into account only limited contact between the ignorant and the spreader. In this study, the contact saturation between the lurker and the spreader is also considered.
(3) The optimal control strategy is proposed, which suppresses the propagation of rumors with the lowest cost and minimizes the number of spreaders in the network.
(4) The comparisons between the Hyper-ILSR model and the ILSR model are shown in numerical simulations to confirm that the Hyper-ILSR model is more realistic than the ILSR model.

The article is organized as follows. In Section 2, hypergraph theories are introduced and a novel Hyper-ILSR rumor propagation model is considered. Section 3 presents the threshold and the equilibrium, and discusses the stability of the equilibrium. Optimal control is given in Section 4. Numerical simulations are provided in Section 5. In Section 6, the conclusion is given.

## 2. Preliminaries and Model Description

In this section, the Hyper-ILSR rumor-spreading model with the forgetting mechanism and saturation incidence rate is considered. Hypergraph theories are presented to reflect the higher-order interactions in rumor dissemination. In hypergraph H(V,E), *V* is the vertex set and *E* is the hyperedge set. The hyperedge is the improvement of the edge, which can be composed of any number of vertices. Similar to the degree, the hyperdegree is the number of hyperedges containing the vertex.

**Figure 1:**
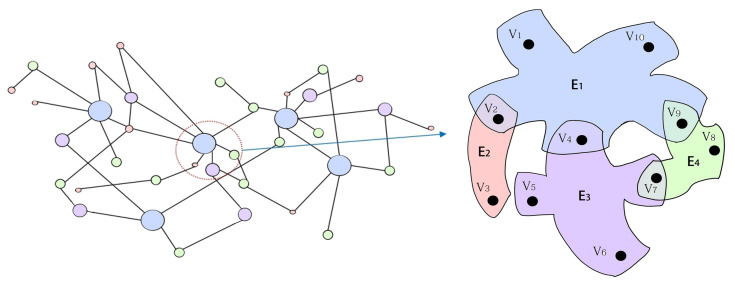
A hypergraph.

In this study, the hyperdegree is represented as the vector. Take WeChat for example, if user U has 60 friends, two groups of 4, and five groups of 30, then the hyperdegree of U is 67, which can be shown as [60, 0, 2, 0, ⋯, 0, 5]. The number of components of the vector depends on the number of nodes contained by the maximum hyperedge in the hypergraph. Figure 1 shows a simple hypergraph. The largest hyperedge contains 5 nodes and the hyperdegree of $V_{5}$ is represented by [0, 0, 1, 0].

The Hyper-ILSR model consists of $I_{K_{i}}\left(t\right)$ (ignorants), $L_{K_{i}}\left(t\right)$ (lurkers), $S_{K_{i}}\left(t\right)$ (spreaders), and $R_{K_{i}}\left(t\right)$ (recovered individuals), denoting the densities of different groups with hyperdegree $K_{i}$ at time *t*. Ignorants have never heard the rumor and they may propagate the rumor after hearing it. Lurkers have known the rumor but hesitate to transmit it. Spreaders mean that the people who spread the rumor. Recovered individuals have heard the rumor but do not propagate it.

**Figure 2:**
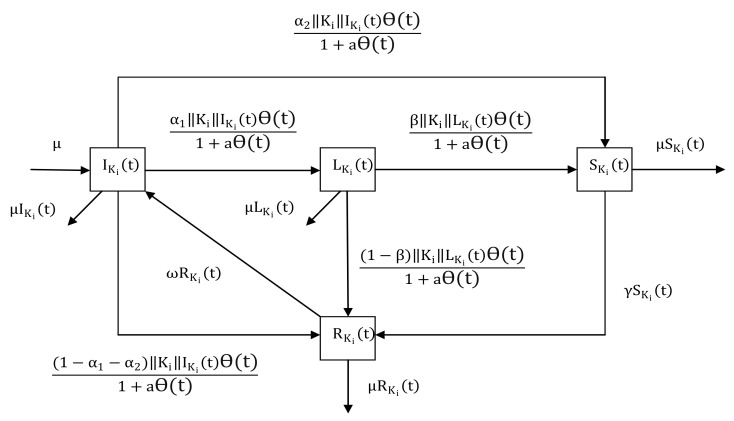
Structure of the Hyper-ILSR rumor propagation process.

As shown in Figure 2, the transition rules can be summarized as follows:

1. If ignorants receive the rumor from spreaders, then they become lurkers, spreaders, and recovered individuals with probability $\alpha_{1}$, $\alpha_{2}$, and $1-\alpha_{1}-\alpha_{2}$, respectively.
2. After lurkers hear the rumor from spreaders, they become spreaders with probability β or recovered individuals with probability 1−β.
3. A spreader knows the truth or loses interest in propagating the rumor, then stops spreading the rumor with probability γ.
4. After a period of time, a recovered individual will become an ignorant with probability ω because of forgetting the rumor.

We assume that the immigration rate is equal to the removal rate, represented by μ. Then, the Hyper-ILSR model in heterogeneous networks can be presented by:

(1) $$\left\{\begin{array}{cc} \frac{dI_{K_{i}}\left(t\right)}{dt}=\; & \mu -\frac{\left|\right|K_{i}\left|\right|I_{K_{i}}\left(t\right)\Theta \left(t\right)}{1+a\Theta (t)}+\omega R_{K_{i}}\left(t\right)-\mu I_{K_{i}}\left(t\right),\\ \frac{dL_{K_{i}}\left(t\right)}{dt}=\; & \frac{\alpha_{1}\left|\right|K_{i}\left|\right|I_{K_{i}}\left(t\right)\Theta \left(t\right)}{1+a\Theta (t)}-\frac{\left|\right|K_{i}\left|\right|L_{K_{i}}\left(t\right)\Theta \left(t\right)}{1+a\Theta (t)}-\mu L_{K_{i}}\left(t\right),\\ \frac{dS_{K_{i}}\left(t\right)}{dt}=\; & \frac{\alpha_{2}\left|\right|K_{i}\left|\right|I_{K_{i}}\left(t\right)\Theta \left(t\right)}{1+a\Theta (t)}+\frac{\beta ||K_{i}\left|\right|L_{K_{i}}\left(t\right)\Theta \left(t\right)}{1+a\Theta (t)}-\left(\gamma +\mu \right)S_{K_{i}}\left(t\right),\\ \frac{dR_{K_{i}}\left(t\right)}{dt}=\; & \frac{\left(1-\alpha_{1}-\alpha_{2}\right)\left|\right|K_{i}\left|\right|I_{K_{i}}\left(t\right)\Theta \left(t\right)}{1+a\Theta (t)}+\frac{\left(1-\beta \right)||K_{i}\left|\right|L_{K_{i}}\left(t\right)\Theta \left(t\right)}{1+a\Theta (t)}\\ \; & +\gamma S_{K_{i}}\left(t\right)-\left(\omega +\mu \right)R_{K_{i}}\left(t\right), \end{array}\right.$$

where $\Theta \left(t\right)=\frac{1}{\langle ||K_{i}||\rangle }\sum_{i=1}^{n}\varphi \left(K_{i}\right)P\left(K_{i}\right)S_{K_{i}}\left(t\right)$, denoting the possibility that any given hyperedge is connected to a spreader.

In model (1), $K_{i}$ denotes the hyperdegree that is ranked *i*. Hyperdegree $K_{i}=\left[k_{i}^{\left(2\right)},k_{i}^{\left(3\right)},\cdots ,k_{i}^{\left(Q\right)}\right]$, $k_{i}^{\left(q\right)}$ represents the *q*th order degree, which refers to the quantity of hyperedges of size *q* containing the individual with hyperdegree $K_{i}$, q=2,3,⋯,Q [30]. *Q* is the quantity of nodes in the maximum hyperedge. $\varphi \left(K_{i}\right)=\sum_{q=2}^{Q}\beta_{q}\frac{k_{i}^{\left(q\right)}}{q-1}$, indicating the infectivity of the propagation of rumors by spreaders with the hyperdegree $K_{i}$. $\beta_{q}$ is the probability that a hyperedge of size *q* infects the ignorant. $\left|\right|K_{i}\left|\right|=\sum_{q=2}^{Q}k_{i}^{\left(q\right)}$[31]. Compare the different hyperdegrees starting from the 2nd order degree, then sort them in ascending order, and if they are the same, compare the third degree. For example, [61, 3, 0,⋯, 0] is behind [60, 3, 0,⋯, 0], and [51, 2, 3, 0,⋯, 0] is behind [51, 2, 2, 0,⋯, 0], and [53, 3, 0, 2, 1,⋯, 0] is in front of [53, 3, 2, 0, 1,⋯, 0].

 

**Remark** **1.** *In general, $\Theta \left(t\right)=\sum_{i=1}^{n}\frac{\varphi \left(K_{i}\right)P\left(K_{i}|K_{j}\right)S_{K_{i}}\left(t\right)}{\left|\right|K_{i}\left|\right|}$, where $P(K_{i}|K_{j})$ denotes the conditional probability. Considering the uncorrelated network,*

$$\Theta \left(t\right)=\frac{1}{\langle ||K_{i}||\rangle }\sum_{i=1}^{n}\varphi \left(K_{i}\right)P\left(K_{i}\right)S_{K_{i}}\left(t\right),$$

*where $P(K_{i})$ is the hyperdegree distribution [31].*

 

**Remark** **2.** *There are hyperedges of size 2, 3, 4, and 5 in the hypergraph in Figure 1, then Q=5. $V_{3}$ is only contained in $E_{2}$, and $E_{2}$ consists of two nodes. Thus, the second degree of $V_{3}$ is 1 and the other degrees are 0. The hyperdegree of $V_{3}$ is represented by [1, 0, 0, 0]. Similarly, the hyperdegree of other nodes can be expressed as $K_{V_{1}}=\left[0,0,0,1\right]$, $K_{V_{2}}=\left[1,0,0,1\right]$, $K_{V_{4}}=\left[0,0,1,1\right]$, $K_{V_{5}}=\left[0,0,1,0\right]$, $K_{V_{6}}=\left[0,0,1,0\right]$, $K_{V_{7}}=\left[0,1,1,0\right]$, $K_{V_{8}}=\left[0,1,0,0\right]$, $K_{V_{9}}=\left[0,1,0,1\right]$, and $K_{V_{10}}=\left[0,0,0,1\right]$. Put these hyperdegrees in ascending order and ignore the same hyperdegree. Then $K_{1}=\left[0,0,0,1\right]$, $K_{2}=\left[0,0,1,0\right]$, $K_{3}=\left[0,0,1,1\right]$, $K_{4}=\left[0,1,0,0\right]$, $K_{5}=\left[0,1,0,1\right]$, $K_{6}=\left[0,1,1,0\right]$, $K_{7}=\left[1,0,0,0\right]$, and $K_{8}=\left[1,0,0,1\right]$.*

In model (1), the initial conditions are defined as $I_{K_{i}}\left(0\right)>0$, $L_{K_{i}}\left(0\right)\geq 0$, $S_{K_{i}}\left(0\right)\geq 0$, $R_{K_{i}}\left(0\right)\geq 0$ and Θ(0)>0.

The feasible region is

$\Gamma =\{\left(I_{K_{i}}\left(t\right),L_{K_{i}}\left(t\right),S_{K_{i}}\left(t\right),R_{K_{i}}\left(t\right)\right)\in R_{+}^{4n}|I_{K_{i}}\left(t\right)+L_{K_{i}}\left(t\right)+S_{K_{i}}\left(t\right)+R_{K_{i}}\left(t\right)=1,$ i=1,2,⋯,n}.

Next, positivity of the solutions is discussed.

 

**Lemma** **1.** *Let $\left\{\left(I_{K_{1}}\left(t\right),L_{K_{1}}\left(t\right),S_{K_{1}}\left(t\right),R_{K_{1}}\left(t\right)\right),\cdots ,\left(I_{K_{n}}\left(t\right),L_{K_{n}}\left(t\right),S_{K_{n}}\left(t\right),R_{K_{n}}\left(t\right)\right)\right\}$ be the solution of system (1) with the initial conditions, then for all t>0, one can obtain that Θ(t)>0 and $0<I_{K_{i}}\left(t\right),L_{K_{i}}\left(t\right),S_{K_{i}}\left(t\right),R_{K_{i}}\left(t\right)<1$, where i=1,2,⋯,n.*

 

**Proof.**  Proved in Appendix A. □

## 3. Dynamical Analysis

In this section, the equilibrium points and the basic reproduction number are obtained, and the stability of the equilibrium points is discussed.

The equilibrium points are calculated as follows.

(2) $$\left\{\begin{array}{c} \mu -\frac{\left|\right|K_{i}\left|\right|I_{K_{i}}^{*}\Theta^{*}}{1+a\Theta^{*}}+\omega R_{K_{i}}^{*}-\mu I_{K_{i}}^{*}=0,\\ \frac{\alpha_{1}\left|\right|K_{i}\left|\right|I_{K_{i}}^{*}\Theta^{*}}{1+a\Theta^{*}}-\frac{\left|\right|K_{i}\left|\right|L_{K_{i}}^{*}\Theta^{*}}{1+a\Theta^{*}}-\mu L_{K_{i}}^{*}=0,\\ \frac{\alpha_{2}\left|\right|K_{i}\left|\right|I_{K_{i}}^{*}\Theta^{*}}{1+a\Theta^{*}}+\frac{\beta ||K_{i}\left|\right|L_{K_{i}}^{*}\Theta^{*}}{1+a\Theta^{*}}-\left(\gamma +\mu \right)S_{K_{i}}^{*}=0,\\ \frac{\left|\right|K_{i}\left|\right|\Theta^{*}\left(\left(1-\alpha_{1}-\alpha_{2}\right)I_{K_{i}}^{*}+\left(1-\beta \right)L_{K_{i}}^{*}\right)}{1+a\Theta^{*}}+\gamma S_{K_{i}}^{*}-\left(\omega +\mu \right)R_{K_{i}}^{*}=0, \end{array}\right.$$

where $\Theta^{*}=\frac{1}{\langle ||K_{i}||\rangle }\sum_{i=1}^{n}\varphi \left(K_{i}\right)P\left(K_{i}\right)S_{K_{i}}^{*}.$

Obviously, $E^{0}=\left(1,0,0,0\right)$ is one equilibrium point of Equation (2). Moreover, from Equation (2), one has

(3) $$\left\{\begin{array}{cc} \; & I_{K_{i}}^{*}=\frac{\left(\gamma +\mu \right)\left(1+a\Theta^{*}\right)(\Theta^{*}\left|\right|K_{i}\left|\right|+\mu \left(1+a\Theta^{*}\right))\left(\omega +\mu \right)}{(\Theta^{*}\left|\right|K_{i}\left|\right|+\mu \left(1+a\Theta^{*}\right))A_{1}+\omega \alpha_{1}\left|\right|K_{i}\left|\right|\Theta^{*}A_{2}},\\ \; & L_{K_{i}}^{*}=\frac{\alpha_{1}\left|\right|K_{i}\left|\right|\Theta^{*}\left(\gamma +\mu \right)\left(1+a\Theta^{*}\right)\left(\omega +\mu \right)}{(\Theta^{*}\left|\right|K_{i}\left|\right|+\mu \left(1+a\Theta^{*}\right))A_{1}+\omega \alpha_{1}\left|\right|K_{i}\left|\right|\Theta^{*}A_{2}},\\ \; & S_{K_{i}}^{*}=\frac{((\Theta^{*}\left|\right|K_{i}\left|\right|+\mu \left(1+a\Theta^{*}\right))\alpha_{2}\left|\right|K_{i}\left|\right|\Theta^{*}+\alpha_{1}\beta ||K_{i}{\left|\right|}^{2}\Theta^{*2})\left(\omega +\mu \right)}{(\Theta^{*}\left|\right|K_{i}\left|\right|+\mu \left(1+a\Theta^{*}\right))A_{1}+\omega \alpha_{1}\left|\right|K_{i}\left|\right|\Theta^{*}A_{2}},\\ \; & R_{K_{i}}^{*}=1-I_{K_{i}}^{*}-L_{K_{i}}^{*}-S_{K_{i}}^{*}, \end{array}\right.$$

where $A_{1}=\omega \left(\gamma +\mu \right)\left(1+a\Theta^{*}\right)+\omega \alpha_{2}\left|\right|K_{i}\left|\right|\Theta^{*}+\left(\gamma +\mu \right)\left(|\right|K_{i}\left|\right|\Theta^{*}+\mu \left(1+a\Theta^{*}\right))$, and $A_{2}=\left(\gamma +\mu \right)\left(1+a\Theta^{*}\right)+\beta \Theta^{*}$.

By calculating the next generation for the model (1), one obtains the basic reproduction number $R_{0}=\frac{1}{\langle ||K_{i}||\rangle }\sum_{i=1}^{n}\varphi \left(K_{i}\right)P\left(K_{i}\right)\frac{\alpha_{2}\left|\right|K_{i}\left|\right|}{\gamma +\mu }.$

 

**Remark** **3.** *The effect of different parameters on $R_{0}$ can be obtained from the expression of $R_{0}$. By calculation, one has*

$$\frac{\partial R_{0}}{\partial \alpha_{2}}=\frac{1}{\langle ||K_{i}||\rangle }\sum_{i=1}^{n}\varphi \left(K_{i}\right)P\left(K_{i}\right)\frac{\left|\right|K_{i}\left|\right|}{\gamma +\mu }>0,$$

$$\frac{\partial R_{0}}{\partial \gamma }=-\frac{1}{\langle ||K_{i}||\rangle }\sum_{i=1}^{n}\varphi \left(K_{i}\right)P\left(K_{i}\right)\frac{\alpha_{2}\left|\right|K_{i}\left|\right|}{{\left(\gamma +\mu \right)}^{2}}<0,$$

$$\frac{\partial R_{0}}{\partial \mu }=-\frac{1}{\langle ||K_{i}||\rangle }\sum_{i=1}^{n}\varphi \left(K_{i}\right)P\left(K_{i}\right)\frac{\alpha_{2}\left|\right|K_{i}\left|\right|}{{\left(\gamma +\mu \right)}^{2}}<0.$$

*The results suggest that $R_{0}$ increases as $\alpha_{2}$ increases. Moreover, if γ and μ become bigger, then $R_{0}$ becomes smaller. In the Hyper-ILSR model and ILSR model, the above parameters have the same influence.*

 

**Remark** **4.** *Compared with the degree in the general ILSR model, the effect of hyperdegree on $R_{0}$ is less obvious, which is similar to the result in [31].*

 

Next, the stability of equilibrium is studied. Based on the analysis, it can be judged whether the rumor will disappear or persist.

 

**Theorem** **1.** *When $R_{0}<1$, rumor-free equilibrium point $E^{0}$ is locally asymptotically stable, and unstable when $R_{0}>1$.*

 

**Proof.**  Proved in Appendix B. □

 

**Theorem** **2.** *If $R_{0}<1$, the rumor-free equilibrium point $E^{0}$ is globally asymptotically stable.*

 

**Proof.**  Proved in Appendix C. □

 

**Remark** **5.** *If we replace saturation incidence with linear incidence, that is, considering a=0, the above theorems still hold (similar to Ref. [13]). The model (1) changes to*

$$\left\{\begin{array}{cc} \frac{dI_{K_{i}}\left(t\right)}{dt}=\; & \mu -\left|\right|K_{i}\left|\right|I_{K_{i}}\left(t\right)\Theta \left(t\right)+\omega R_{K_{i}}\left(t\right)-\mu I_{K_{i}}\left(t\right),\\ \frac{dL_{K_{i}}\left(t\right)}{dt}=\; & \alpha_{1}\left|\right|K_{i}\left|\right|I_{K_{i}}\left(t\right)\Theta \left(t\right)-\left|\right|K_{i}\left|\right|L_{K_{i}}\left(t\right)\Theta \left(t\right)-\mu L_{K_{i}}\left(t\right),\\ \frac{dS_{K_{i}}\left(t\right)}{dt}=\; & \alpha_{2}\left|\right|K_{i}\left|\right|I_{K_{i}}\left(t\right)\Theta \left(t\right)+\beta ||K_{i}\left|\right|L_{K_{i}}\left(t\right)\Theta \left(t\right)-\left(\gamma +\mu \right)S_{K_{i}}\left(t\right),\\ \frac{dR_{K_{i}}\left(t\right)}{dt}=\; & \left(1-\alpha_{1}-\alpha_{2}\right)\left|\right|K_{i}\left|\right|I_{K_{i}}\left(t\right)\Theta \left(t\right)+\left(1-\beta \right)\left|\right|K_{i}\left|\right|L_{K_{i}}\left(t\right)\Theta \left(t\right)\\ \; & +\gamma S_{K_{i}}\left(t\right)-\left(\omega +\mu \right)R_{K_{i}}\left(t\right). \end{array}\right.$$

Then the stability of $E^{*}\left(I_{K_{i}}^{*},L_{K_{i}}^{*},S_{K_{i}}^{*},R_{K_{i}}^{*}\right)$ is investigated.

 

**Theorem** **3.** If $R_{0}>1$, the equilibrium point $E^{*}$ is globally asymptotically stable.

 

**Proof.**  Proved in Appendix D. □

 

**Remark** **6.** *Hyperdegree $K_{i}=\left[k_{i}^{\left(2\right)},k_{i}^{\left(3\right)},\cdots ,k_{i}^{\left(Q\right)}\right]$, considering Q=2, then $K_{i}=k_{i}^{\left(2\right)}=k$ and $\left|\right|K_{i}\left|\right|=k$, where k is the degree of nodes. Thus, the Hyper-ILSR propagation model becomes the general ILSR model when Q=2. As follows:*

$$\left\{\begin{array}{cc} \frac{dI_{K_{i}}\left(t\right)}{dt}=\; & \mu -\frac{kI_{K_{i}}\left(t\right)\Theta \left(t\right)}{1+a\Theta (t)}+\omega R_{K_{i}}\left(t\right)-\mu I_{K_{i}}\left(t\right),\\ \frac{dL_{K_{i}}\left(t\right)}{dt}=\; & \frac{\alpha_{1}kI_{K_{i}}\left(t\right)\Theta \left(t\right)}{1+a\Theta (t)}-\frac{kL_{K_{i}}\left(t\right)\Theta \left(t\right)}{1+a\Theta (t)}-\mu L_{K_{i}}\left(t\right),\\ \frac{dS_{K_{i}}\left(t\right)}{dt}=\; & \frac{\alpha_{2}kI_{K_{i}}\left(t\right)\Theta \left(t\right)}{1+a\Theta (t)}+\frac{\beta kL_{K_{i}}\left(t\right)\Theta \left(t\right)}{1+a\Theta (t)}-\left(\gamma +\mu \right)S_{K_{i}}\left(t\right),\\ \frac{dR_{K_{i}}\left(t\right)}{dt}=\; & \frac{\left(1-\alpha_{1}-\alpha_{2}\right)kI_{K_{i}}\left(t\right)\Theta \left(t\right)}{1+a\Theta (t)}+\frac{\left(1-\beta \right)kL_{K_{i}}\left(t\right)\Theta \left(t\right)}{1+a\Theta (t)}\\ \; & +\gamma S_{K_{i}}\left(t\right)-\left(\omega +\mu \right)R_{K_{i}}\left(t\right). \end{array}\right.$$

 

**Remark** **7.** *The above theorems are discussed in the vector form of hyperdegree. When the hyperdegrees are numbers, the above theorems still hold.*

## 4. Optimal Control

Optimal control is used to reduce the quantity of spreaders with minimum cost. In reality, when a rumor is generated and widely spread, the official will take some measures to curb the spread of the rumor, and the intensity of these measures depends on the spread scope of the rumor. The optimal control strategy can help to find the appropriate control intensity with low cost. In this section, considering the optimal control strategy for model (1), the control system is given by

(4) $$\left\{\begin{array}{cc} \frac{dI_{K_{i}}\left(t\right)}{dt}=\; & \mu -\frac{\left|\right|K_{i}\left|\right|I_{K_{i}}\left(t\right)\Theta \left(t\right)}{1+a\Theta (t)}+\omega R_{K_{i}}\left(t\right)-\mu I_{K_{i}}\left(t\right),\\ \frac{dL_{K_{i}}\left(t\right)}{dt}=\; & \frac{\alpha_{1}\left|\right|K_{i}\left|\right|I_{K_{i}}\left(t\right)\Theta \left(t\right)}{1+a\Theta (t)}-\frac{\left|\right|K_{i}\left|\right|L_{K_{i}}\left(t\right)\Theta \left(t\right)}{1+a\Theta (t)}-\mu L_{K_{i}}\left(t\right)\\ \; & +bu_{K_{i}}\left(t\right)S_{K_{i}}\left(t\right),\\ \frac{dS_{K_{i}}\left(t\right)}{dt}=\; & \frac{\alpha_{2}\left|\right|K_{i}\left|\right|I_{K_{i}}\left(t\right)\Theta \left(t\right)}{1+a\Theta (t)}+\frac{\beta ||K_{i}\left|\right|L_{K_{i}}\left(t\right)\Theta \left(t\right)}{1+a\Theta (t)}-\left(\gamma +\mu \right)S_{K_{i}}\left(t\right)\\ \; & -u_{K_{i}}\left(t\right)S_{K_{i}}\left(t\right),\\ \frac{dR_{K_{i}}\left(t\right)}{dt}=\; & \frac{\left(1-\alpha_{1}-\alpha_{2}\right)\left|\right|K_{i}\left|\right|I_{K_{i}}\left(t\right)\Theta \left(t\right)}{1+a\Theta (t)}+\frac{\left(1-\beta \right)||K_{i}\left|\right|L_{K_{i}}\left(t\right)\Theta \left(t\right)}{1+a\Theta (t)}\\ \; & +\gamma S_{K_{i}}\left(t\right)-\left(\omega +\mu \right)R_{K_{i}}\left(t\right)+\left(1-b\right)u_{K_{i}}\left(t\right)S_{K_{i}}\left(t\right), \end{array}\right.$$

where $u_{K_{i}}\left(t\right)$ represents the control strengths.

The objective function is considered as

$$G\left(u\right)=\int_{0}^{T}\sum_{i=1}^{n}\left\{S_{K_{i}}\left(t\right)+\frac{A_{K_{i}}}{2}u_{K_{i}}^{2}\left(t\right)\right\}dt,$$

where $A_{K_{i}}$ is the weight coefficient.

Define the Lagrangian as follows:

$$L\left(S_{K_{i}}\left(t\right),u_{K_{i}}\left(t\right)\right)=\sum_{i=1}^{n}\left\{S_{K_{i}}\left(t\right)+\frac{A_{K_{i}}}{2}u_{K_{i}}^{2}\left(t\right)\right\},$$

and take the Hamiltonian function:

(5) $$\begin{array}{cc} \; & H(I_{K_{i}}\left(t\right),L_{K_{i}}\left(t\right),S_{K_{i}}\left(t\right),R_{K_{i}}\left(t\right),u_{K_{i}}\left(t\right),\lambda_{1K_{i}}\left(t\right),\lambda_{2K_{i}}\left(t\right),\lambda_{3K_{i}}\left(t\right),\lambda_{4K_{i}}\left(t\right))\\ =\; & L(S_{K_{i}}\left(t\right),u_{K_{i}}\left(t\right))\\ & +\sum_{i=1}^{n}\{\lambda_{1K_{i}}\left(t\right)\frac{dI_{K_{i}}\left(t\right)}{dt}+\lambda_{2K_{i}}\left(t\right)\frac{dL_{K_{i}}\left(t\right)}{dt}+\lambda_{3K_{i}}\left(t\right)\frac{dS_{K_{i}}\left(t\right)}{dt}\\ \; & +\lambda_{4K_{i}}\left(t\right)\frac{dR_{K_{i}}\left(t\right)}{dt}\}, \end{array}$$

where $\lambda_{1K_{i}}\left(t\right)$, $\lambda_{2K_{i}}\left(t\right)$, $\lambda_{3K_{i}}\left(t\right)$, and $\lambda_{4K_{i}}\left(t\right)$ are the adjoint functions.

The following theorem can be obtained by applying Pontryagin’s Minimum principle [32,33].

 

**Theorem** **4.** The optimal solution is ($I_{K_{i}}^{*}\left(t\right)$, $L_{K_{i}}^{*}\left(t\right)$, $S_{K_{i}}^{*}\left(t\right)$, $R_{K_{i}}^{*}\left(t\right)$) with optimal control $u_{K_{i}}^{*}\left(t\right)$ for the model (4). Then, the adjoint functions $\lambda_{1K_{i}}\left(t\right)$, $\lambda_{2K_{i}}\left(t\right)$, $\lambda_{3K_{i}}\left(t\right)$, and $\lambda_{4K_{i}}\left(t\right)$ satisfying

(6) $$\left\{\begin{array}{cc} \frac{d\lambda_{1K_{i}}\left(t\right)}{dt}= & -\lambda_{1K_{i}}\left(t\right)\left(\frac{\left|\right|K_{i}\left|\right|\Theta^{*}\left(t\right)}{1+a\Theta^{*}\left(t\right)}+\mu \right)-\lambda_{2K_{i}}\left(t\right)\frac{\alpha_{1}\left|\right|K_{i}\left|\right|\Theta^{*}\left(t\right)}{1+a\Theta^{*}\left(t\right)}\\ \; & -\lambda_{3K_{i}}\left(t\right)\frac{\alpha_{2}\left|\right|K_{i}\left|\right|\Theta^{*}\left(t\right)}{1+a\Theta^{*}\left(t\right)}-\lambda_{4K_{i}}\left(t\right)\frac{\left(1-\alpha_{1}-\alpha_{2}\right)\left|\right|K_{i}\left|\right|\Theta^{*}\left(t\right)}{1+a\Theta^{*}\left(t\right)},\\ \frac{d\lambda_{2K_{i}}\left(t\right)}{dt}= & -\lambda_{2K_{i}}\left(t\right)\left(\frac{\left|\right|K_{i}\left|\right|\Theta^{*}\left(t\right)}{1+a\Theta^{*}\left(t\right)}+\mu \right)-\lambda_{3K_{i}}\left(t\right)\frac{\beta ||K_{i}\left|\right|\Theta^{*}\left(t\right)}{1+a\Theta^{*}\left(t\right)}\\ \; & -\lambda_{4K_{i}}\left(t\right)\frac{\left(1-\beta \right)||K_{i}\left|\right|\Theta^{*}\left(t\right)}{1+a\Theta^{*}\left(t\right)},\\ \frac{d\lambda_{3K_{i}}\left(t\right)}{dt}=\; & \lambda_{1K_{i}}\left(t\right)\frac{\left|\right|K_{i}\left|\right|I_{K_{i}}^{*}\left(t\right)\Psi \left(i\right)}{{\left(1+a\Theta^{*}\left(t\right)\right)}^{2}}-\lambda_{2K_{i}}\left(t\right)\frac{\alpha_{1}\left|\right|K_{i}\left|\right|I_{K_{i}}^{*}\left(t\right)\Psi \left(i\right)}{{\left(1+a\Theta^{*}\left(t\right)\right)}^{2}}\\ \; & +\lambda_{2K_{i}}\left(t\right)\frac{\left|\right|K_{i}\left|\right|L_{K_{i}}^{*}\left(t\right)\Psi \left(i\right)}{{\left(1+a\Theta^{*}\left(t\right)\right)}^{2}}-\lambda_{2K_{i}}\left(t\right)bu_{K_{i}}^{*}\left(t\right)\\ \; & -\lambda_{3K_{i}}\left(t\right)\frac{\alpha_{2}\left|\right|K_{i}\left|\right|I_{K_{i}}^{*}\left(t\right)\Psi \left(i\right)}{{\left(1+a\Theta^{*}\left(t\right)\right)}^{2}}-\lambda_{3K_{i}}\left(t\right)\frac{\beta ||K_{i}\left|\right|L_{K_{i}}^{*}\left(t\right)\Psi \left(i\right)}{{\left(1+a\Theta^{*}\left(t\right)\right)}^{2}}\\ \; & +\left(\gamma +\mu \right)\lambda_{3K_{i}}\left(t\right)+\lambda_{3K_{i}}\left(t\right)u_{K_{i}}^{*}\left(t\right)+W\\ \frac{d\lambda_{4K_{i}}\left(t\right)}{dt} & =-\{\lambda_{1K_{i}}\left(t\right)\omega +\lambda_{4K_{i}}\left(t\right)\left(-\left(\omega +\mu \right)\right)\}, \end{array}\right.$$

with transversality conditions $\lambda_{1K_{i}}\left(T\right)=\lambda_{2K_{i}}\left(T\right)=\lambda_{3K_{i}}\left(T\right)=\lambda_{4K_{i}}\left(T\right)=0$, i=1,2,⋯,n. $W=-\lambda_{4K_{i}}\left(t\right)\frac{\left(1-\alpha_{1}-\alpha_{2}\right)\left|\right|K_{i}\left|\right|I_{K_{i}}^{*}\left(t\right)\Psi \left(i\right)}{{\left(1+a\Theta^{*}\left(t\right)\right)}^{2}}-\gamma \lambda_{4K_{i}}\left(t\right)-\lambda_{4K_{i}}\left(t\right)\frac{\left(1-\beta \right)||K_{i}\left|\right|L_{K_{i}}^{*}\left(t\right)\Psi \left(i\right)}{{\left(1+a\Theta^{*}\left(t\right)\right)}^{2}}-\lambda_{4K_{i}}\left(t\right)\left(1-b\right)u_{K_{i}}^{*}\left(t\right)$. Furthermore, the optimal control

$$u_{K_{i}}^{*}\left(t\right)=\max\left\{\min\left\{\frac{\left(-\lambda_{2K_{i}}\left(t\right)b+\lambda_{3K_{i}}\left(t\right)-\left(1-b\right)\lambda_{4K_{i}}\left(t\right)\right)S_{K_{i}}^{*}\left(t\right)}{A_{K_{i}}},1\right\},0\right\}.$$

 

**Proof.**  Proved in Appendix E. □

## 5. Numerical Simulations

To demonstrate the validity of the above analysis, some numerical simulations are presented. The study is based on the network with hyperdegree distribution $P(K_{i})$: $P\left(K_{i}\right)\propto \left(m+1\right)!||K_{i}{\left|\right|}^{-2-m}$, where *m* is the minimum value of $\left|\right|K_{i}\left|\right|$. Take $\left|\right|K_{i}\left|\right|$ from 1 to 200, we can obtain $\langle ||K_{i}||\rangle =3.27$ and $\langle ||K_{i}|{|}^{2}\rangle =11.75$. Furthermore, the average value of $\varphi (K_{i})$ is represented by ϕ.

### 5.1. Stability of $E^{0}$

**Figure 3:**
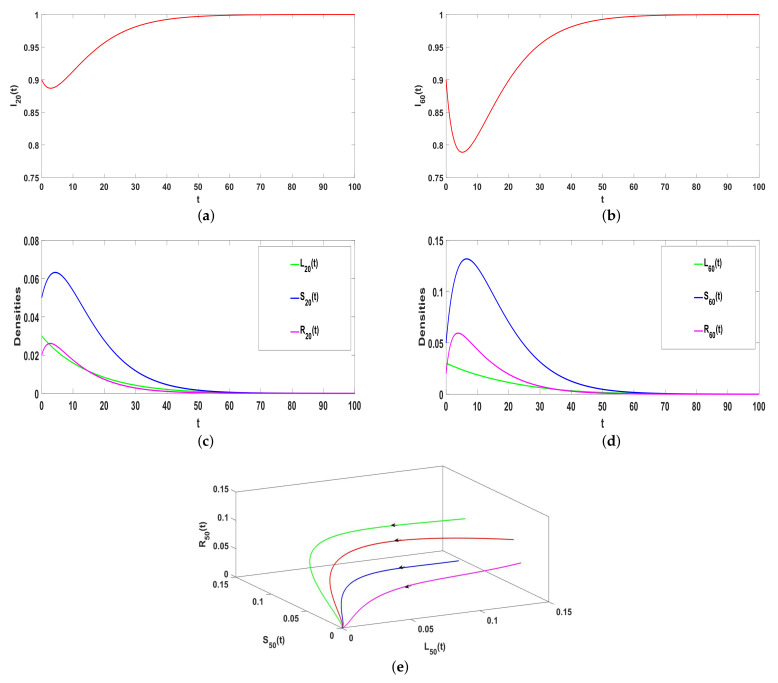
(**a**–**e**) The stability of Hyper-ILSR model when $R_{0}<1$.

Take ϕ=0.04, a=0.05, $\alpha_{1}=0.05$, $\alpha_{2}=0.5$, β=0.63, γ=0.05, ω=0.375, and μ=0.08, respectively, thus $R_{0}<1$. Based on Theorem 1, $E^{0}$ is locally asymptotically stable, which can be confirmed by Figure 3a–d. In addition, $E^{0}$ is globally asymptotic stable, which can be shown in Figure 3e. As time goes on, rumors will eventually disappear.

 

**Figure 4:**
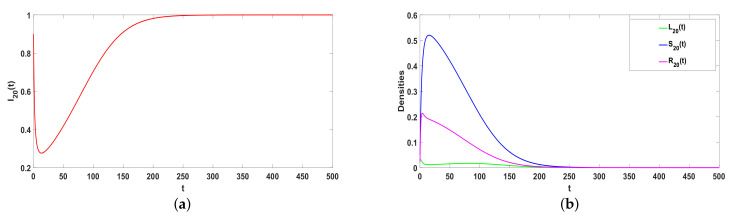
(**a**,**b**) The stability of ILSR model when $R_{0}<1$.

**Remark** **8.** *The general ILSR model with saturation incidence rate can be regarded as the Hyper-ILSR model when Q=2, that is $\varphi \left(K_{i}\right)=\beta_{2}k_{i}^{\left(2\right)}$. Take a=0.05, $\alpha_{1}=0.05$, $\alpha_{2}=0.5$, β=0.63, γ=0.05, ω=0.375, μ=0.08, and $\beta_{2}=0.31$. Obviously, $R_{0}<1$. Compared with Figure 3a,c and Figure 4 illustrates that the ILSR model takes longer to reach the equilibrium. Because the simple graph only considers the spread of point-to-point, the hypergraph also considers the spread of groups. In reality, most rumors spread through the Internet and spread quickly. After the rumors are clarified, the number of rumor spreaders decreases rapidly and the rumors gradually disappear. Thus, compared with the two models, the Hyper-ILSR model is more realistic.*

### 5.2. Stability of $E^{*}$

**Figure 5:**
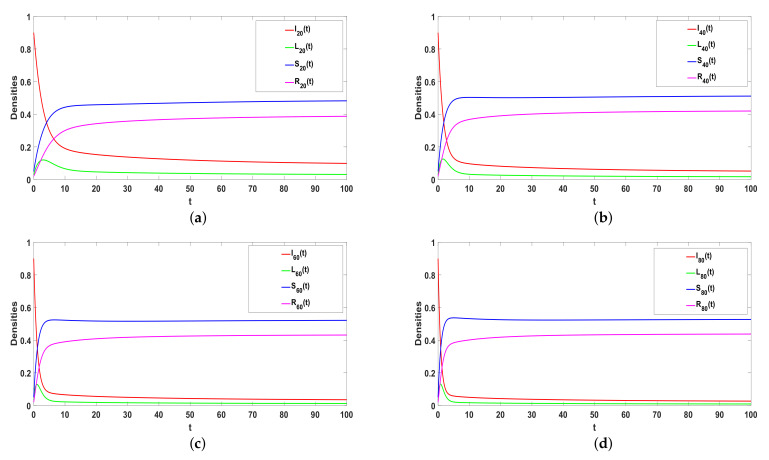

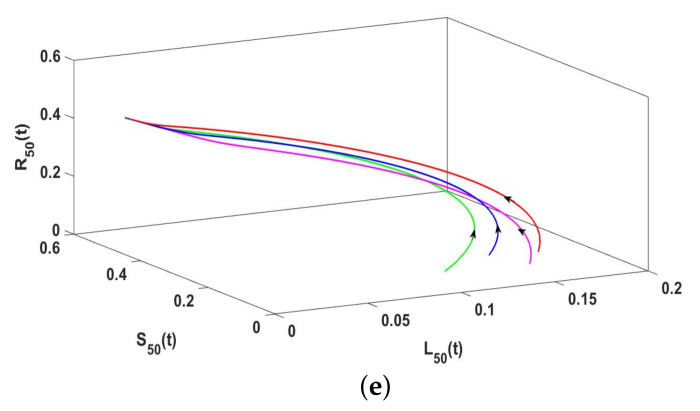
(**a**–**e**) The stability of Hyper-ILSR model when $R_{0}>1$.

Take ϕ=0.37, a=0.05, $\alpha_{1}=0.35$, $\alpha_{2}=0.5$, β=0.26, γ=0.0075, ω=0.001, μ=0.08, then $R_{0}>1$. From Theorem 3, $E^{*}$ is globally asymptotic stable, which is consistent with Figure 5. In addition, Figure 5a–d illustrates the higher the hyperdegree is, the faster rumors reach equilibrium. The greater the individual’s hyperdegree, the greater the probability of contacting and spreading rumors, the faster and wider the spread of rumors in social networks, and the faster the rumors can stabilize.

 

**Figure 6:**
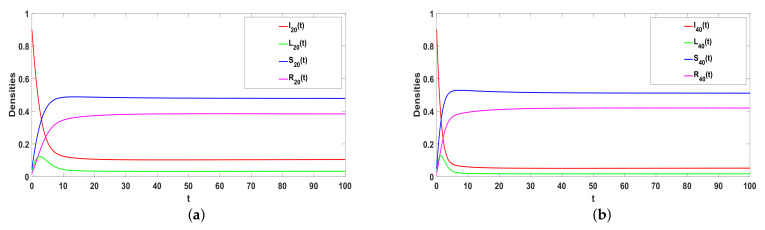
(**a**,**b**) The stability of ILSR model when $R_{0}>1$.

**Remark** **9.** *Considering Q=2. Take a=0.05, $\alpha_{1}=0.35$, $\alpha_{2}=0.5$, β=0.26, γ=0.0075, ω=0.001, μ=0.08, and $\beta_{2}=0.31$. Figure 6 shows the greater the degree, the faster the equilibrium will be reached. The larger the degree, that is, the more edges the individual has with other nodes, the greater the probability of contacting and spreading rumors. Thus, the faster and wider the spread range of rumors in the social network, the rumor stabilizes faster in the network.*

### 5.3. Effects of Parameter *A*

**Figure 7:**
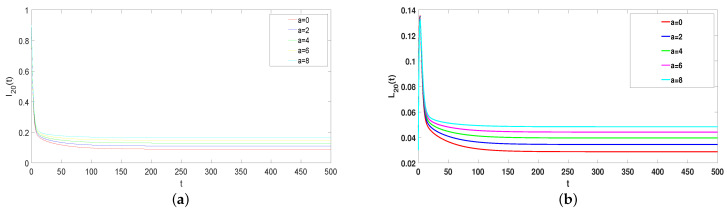

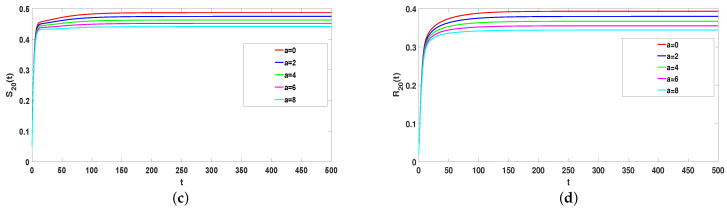
(**a**–**d**) The effects of *a* on rumor spreading ($R_{0}>1$).

The impact of parameter *a* in model (1) will be analyzed in the following. Take ϕ=0.37, $\alpha_{1}=0.35$, $\alpha_{2}=0.5$, β=0.26, γ=0.0075, ω=0.001, and μ=0.08. Choose *a* = 0, 2, 4, 6, and 8. When the system is in equilibrium, Figure 7 shows the value of $I_{20}\left(t\right)$ and $L_{20}\left(t\right)$ increases with increasing *a*, while the value of $S_{20}\left(t\right)$ and $R_{20}\left(t\right)$ decreases. Thus, the rumor can be reduced by increasing the psychological factor. In real life, the government can intensify science popularization or education to increase the effect of psychological factors.

### 5.4. Optimal Control

**Figure 8:**
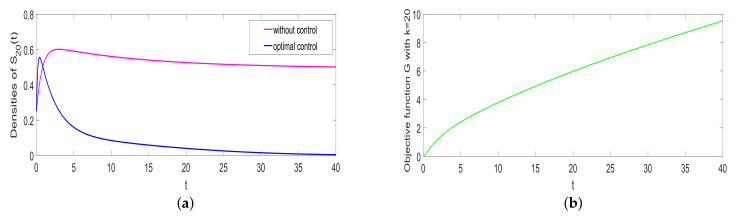
Optimal control (**a**) and control costs (**b**).

Considering the controlled system (4), take ϕ=0.37, a=0.05, $\alpha_{1}=0.35$, $\alpha_{2}=0.5$, β=0.26, γ=0.0075, ω=0.001, μ=0.08, and $A_{K_{i}}=2$. Figure 8a indicates that the densities of rumor spreaders go down to zero under optimal control, which means that the optimal control can control rumor-spreading. Moreover, the cost is shown in Figure 8b.

### 5.5. Model Application

**Figure 9:**
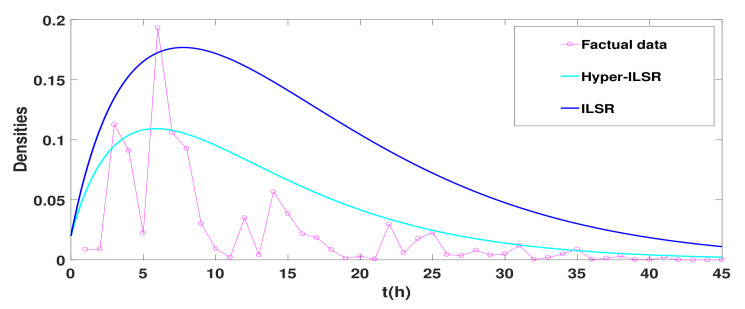
The comparison of Hyper-ILSR and ILSR model with factual data.

Next, an actual rumor case will be introduced to verify the accuracy of the Hyper-ILSR model. On 22 December 2017, a rumor was spread on Sina Weibo about “tourists touching elephant tails in Thailand and causing the leader of the group to be trampled to death”. The data provided by [34] are shown in Table 1. To better show the difference, the number of reprints is converted to density in Figure 9.

Take ϕ=0.13, a=0.05, $\alpha_{1}=0.05$, $\alpha_{2}=0.5$, β=0.93, γ=0.0005, ω=0.475, μ=0.18, and $\beta_{2}=0.13$. Figure 9 shows that the Hyper-ILSR model goes to 0 faster and fits the real data better than the general ILSR model.

## 6. Conclusions

Due to the expansion of the scale of social networks and the increasingly complex relationships between individuals, the rumor model based on a simple graph can no longer fully meet the needs of describing the topology of networks. A simple graph can only describe the interaction between a couple, but cannot show the interaction among multiple individuals. In this study, considering the effect of the higher-order interactions on rumor propagation, a Hyper-ILSR rumor-spreading model with the saturation incidence rate based on hypergraph theories is studied. The hyperdegree can reflect the interactions between individuals and groups. At first, the basic reproduction number $R_{0}$ is calculated. Second, we verify that rumor-free (rumor-prevailing) equilibrium is globally asymptotically stable when $R_{0}<1$ ($R_{0}>1$). Furthermore, optimal control is proposed to reduce spreaders. Finally, numerical simulations demonstrate the impact of parameter *a* on the number of spreaders when the equilibrium is reached. In the future, the influence of age structure and education on the rumor-spreading process will be considered. In addition, more control strategies will be used to suppress the propagation of rumors, such as acquaintance immunization control, pulse control, and event-triggered control.

## Figures and Tables

**Table 1:** The number of reprints.

Time	1 h	2 h	3 h	4 h	5 h	6 h	7 h	8 h	9 h	10 h	11 h
Reprints	85	89	1117	902	220	1914	1050	919	299	92	20
Time	12 h	13 h	14 h	15 h	16 h	17 h	18 h	19 h	20 h	21 h	22 h
Reprints	346	40	562	381	214	182	83	13	31	6	294
Time	23 h	24 h	25 h	26 h	27 h	28 h	29 h	30 h	31 h	32 h	33 h
Reprints	57	176	226	44	34	79	39	50	119	4	20
Time	34 h	35 h	36 h	37 h	38 h	39 h	40 h	41 h	42 h	43 h	44 h
Reprints	48	86	3	13	30	2	2	22	0	0	0

## Data Availability

Not applicable.

## Appendix A. The Proof of Lemma 1

**Proof****.** First, for all t>0, Θ(t)>0 is verified. From model (1), one can obtain

(A1) $$\begin{array}{cc} \Theta^{{}^{\prime }}\left(t\right) & =\frac{1}{\langle ||K_{i}||\rangle }\sum_{i=1}^{n}\varphi \left(K_{i}\right)P\left(K_{i}\right)S_{K_{i}}^{{}^{\prime }}\left(t\right)\\ \; & =\sum_{i=1}^{n}\frac{\varphi \left(K_{i}\right)P\left(K_{i}\right)}{\langle ||K_{i}||\rangle }\left(\frac{\left|\right|K_{i}\left|\right|\Theta \left(t\right)\left(\alpha_{2}I_{K_{i}}\left(t\right)+\beta L_{K_{i}}\left(t\right)\right)}{1+a\Theta (t)}-\left(\gamma +\mu \right)S_{K_{i}}\left(t\right)\right)\\ \; & =\Theta \left(t\right)(\sum_{i=1}^{n}\frac{\varphi \left(K_{i}\right)P\left(K_{i}\right)}{\langle ||K_{i}||\rangle }\left(\frac{\left|\right|K_{i}\left|\right|\left(\alpha_{2}I_{K_{i}}\left(t\right)+\beta L_{K_{i}}\left(t\right)\right)}{1+a\Theta (t)}-\left(\gamma +\mu \right)\right). \end{array}$$

By integrating (A1), one has

$$\Theta \left(t\right)=\Theta \left(0\right)\exp\{\int_{0}^{t}\sum_{i=1}^{n}\frac{\varphi \left(K_{i}\right)P\left(K_{i}\right)}{\langle ||K_{i}||\rangle }\left(\frac{\left|\right|K_{i}\left|\right|\left(\alpha_{2}I_{K_{i}}\left(t\right)+\beta L_{K_{i}}\left(t\right)\right)}{1+a\Theta (t)}\right)dt-\left(\gamma +\mu \right)t\}.$$

Due to Θ(0)>0, then for all t>0, Θ(t)>0. Define $g\left(t\right)=\min_{t}\left\{I_{K_{i}}\left(t\right),L_{K_{i}}\left(t\right),S_{K_{i}}\left(t\right),R_{K_{i}}\left(t\right)\right\}$. Suppose there is a sufficient small ς>0 to make $g\left(\varsigma \right)=0,g^{\prime }\left(t\right)<0$ and g(t)>0, where t∈(0,ς). If $g\left(\varsigma \right)=I_{K_{i}}\left(\varsigma \right)=0$, then $L_{K_{i}}\left(\varsigma \right),S_{K_{i}}\left(\varsigma \right),R_{K_{i}}\left(\varsigma \right)\geq 0$. In addition, it has $\frac{dI_{K_{i}}\left(\varsigma \right)}{dt}=\mu +\omega R_{K_{i}}\left(\varsigma \right)>0$ from model (1), that is $I_{K_{i}}\left(\varsigma \right)>I_{K_{i}}\left(t\right)$, which contradicts $I_{K_{i}}\left(t\right)>0=I_{K_{i}}\left(\varsigma \right)$ for t∈(0,ς). Likewise, one has $L_{K_{i}}\left(t\right),S_{K_{i}}\left(t\right),R_{K_{i}}\left(t\right)>0.$ □

## Appendix B. The Proof of Theorem 1

**Proof****.** Model (1) can be written as

(A2) $$\left\{\begin{array}{cc} \frac{dI_{K_{i}}\left(t\right)}{dt}=\; & \mu -\frac{\left|\right|K_{i}\left|\right|I_{K_{i}}\left(t\right)\Theta \left(t\right)}{1+a\Theta (t)}+\omega \left(1-I_{K_{i}}\left(t\right)-L_{K_{i}}\left(t\right)-S_{K_{i}}\left(t\right)\right)\\ \; & -\mu I_{K_{i}}\left(t\right),\\ \frac{dL_{K_{i}}\left(t\right)}{dt}=\; & \frac{\alpha_{1}\left|\right|K_{i}\left|\right|I_{K_{i}}\left(t\right)\Theta \left(t\right)}{1+a\Theta (t)}-\frac{\left|\right|K_{i}\left|\right|L_{K_{i}}\left(t\right)\Theta \left(t\right)}{1+a\Theta (t)}-\mu L_{K_{i}}\left(t\right),\\ \frac{dS_{K_{i}}\left(t\right)}{dt}=\; & \frac{\alpha_{2}\left|\right|K_{i}\left|\right|I_{K_{i}}\left(t\right)\Theta \left(t\right)}{1+a\Theta (t)}+\frac{\beta ||K_{i}\left|\right|L_{K_{i}}\left(t\right)\Theta \left(t\right)}{1+a\Theta (t)}-\left(\gamma +\mu \right)S_{K_{i}}\left(t\right). \end{array}\right.$$

The Jacobian matrix of system (A2) at $E^{0}$ is

$$J\left(E^{0}\right)=\left(\begin{array}{ccc} A_{11} & A_{12} & A_{13}\\ 0 & A_{22} & A_{23}\\ 0 & 0 & A_{33} \end{array}\right),$$

where $A_{11}={\left(\begin{array}{ccc} -\omega -\mu & \cdots & 0\\ \vdots & \ddots & \vdots \\ 0 & \cdots & -\omega -\mu \end{array}\right)}_{n\times n},$ $A_{12}={\left(\begin{array}{ccc} -\omega & \cdots & 0\\ \vdots & \ddots & \vdots \\ 0 & \cdots & -\omega \end{array}\right)}_{n\times n},$

$A_{13}={\left(\begin{array}{ccc} \left|\right|K_{1}\left|\right|\Psi \left(1\right)-\omega & \cdots & \left|\right|K_{1}\left|\right|\Psi \left(n\right)\\ \vdots & \ddots & \vdots \\ \left|\right|K_{n}\left|\right|\Psi \left(1\right) & \cdots & \left|\right|K_{n}\left|\right|\Psi \left(n\right)-\omega \end{array}\right)}_{n\times n},$ $A_{22}={\left(\begin{array}{ccc} -\mu & \cdots & 0\\ \vdots & \ddots & \vdots \\ 0 & \cdots & -\mu \end{array}\right)}_{n\times n},$

$A_{23}={\left(\begin{array}{ccc} \alpha_{1}\left|\right|K_{1}\left|\right|\Psi \left(1\right) & \cdots & \alpha_{1}\left|\right|K_{1}\left|\right|\Psi \left(n\right)\\ \vdots & \ddots & \vdots \\ \alpha_{1}\left|\right|K_{n}\left|\right|\Psi \left(1\right) & \cdots & \alpha_{1}\left|\right|K_{n}\left|\right|\Psi \left(n\right) \end{array}\right)}_{n\times n},$

$$A_{33}={\left(\begin{array}{ccc} \alpha_{2}\left|\right|K_{1}\left|\right|\Psi \left(1\right)-\left(\gamma +\mu \right) & \cdots & \alpha_{2}\left|\right|K_{1}\left|\right|\Psi \left(n\right)\\ \vdots & \ddots & \vdots \\ \alpha_{2}\left|\right|K_{n}\left|\right|\Psi \left(1\right) & \cdots & \alpha_{2}\left|\right|K_{n}\left|\right|\Psi \left(n\right)-\left(\gamma +\mu \right) \end{array}\right)}_{n\times n},$$

and $\Psi \left(i\right)=\frac{\varphi \left(K_{i}\right)P\left(K_{i}\right)}{\langle ||K_{i}||\rangle }$. By calculating, the characteristic equation is

(A3) $${\left(\lambda +\gamma +\mu \right)}^{n-1}{\left(\lambda +\omega +\mu \right)}^{n}{\left(\lambda +\mu \right)}^{n}(\lambda +\gamma +\mu -\sum_{i=1}^{n}\alpha_{2}\left|\right|K_{i}\left||\Psi \left(i\right)\right)=0.$$

Clearly, −γ−μ, −ω−μ, and −μ are negative. Additionally, $\sum_{i=1}^{n}\alpha_{2}\left|\right|K_{i}\left|\right|\Psi \left(i\right)-\gamma -\mu =\left(\gamma +\mu \right)\left(R_{0}-1\right).$ Hence, all roots of (A3) are negative if $R_{0}<1$, and $E^{0}$ is locally asymptotically stable. Additionally, $E^{0}$ is unstable if $R_{0}>1$ based on Routh–Hurwitz criteria. □

## Appendix C. The Proof of Theorem 2

**Proof****.** Consider a Lyapunov function as follows:

$$V\left(t\right)=\frac{1}{2}\Theta^{2}\left(t\right).$$

Then, one has

$$\begin{array}{cc} V^{{}^{\prime }}\left(t\right)=\; & \frac{\Theta (t)}{\langle ||K_{i}||\rangle }\sum_{i=1}^{n}\varphi \left(K_{i}\right)P\left(K_{i}\right)\frac{\alpha_{2}\left|\right|K_{i}\left|\right|I_{K_{i}}\left(t\right)\Theta \left(t\right)+\beta ||K_{i}\left|\right|L_{K_{i}}\left(t\right)\Theta \left(t\right)}{1+a\Theta (t)}\\ \; & -\frac{\Theta (t)}{\langle ||K_{i}||\rangle }\sum_{i=1}^{n}\varphi \left(K_{i}\right)P\left(K_{i}\right)\left(\gamma +\mu \right)S_{K_{i}}\left(t\right)\\ =\; & \frac{\Theta^{2}\left(t\right)}{1+a\Theta }\sum_{i=1}^{n}\varphi \left(K_{i}\right)P\left(K_{i}\right)\frac{\alpha_{2}\left|\right|K_{i}\left|\right|\left(I_{K_{i}}\left(t\right)+\frac{\beta }{\alpha_{2}}L_{K_{i}}\left(t\right)\right)}{\langle ||K_{i}||\rangle }\\ & -\left(\gamma +\mu \right)\Theta^{2}\left(t\right)\\ =\; & \frac{\left(\gamma +\mu \right)\Theta^{2}\left(t\right)}{1+a\Theta }\left(\sum_{i=1}^{n}\varphi \left(K_{i}\right)P\left(K_{i}\right)\frac{\alpha_{2}\left|\right|K_{i}\left|\right|}{\left(\gamma +\mu \right)\langle ||K_{i}||\rangle }-\left(1+a\Theta \right)\right)\\ =\; & \frac{\left(\gamma +\mu \right)\Theta^{2}\left(t\right)}{1+a\Theta }\left(R_{0}-\left(1+a\Theta \right)\right)\\ \leq & \;0. \end{array}$$

Following LaSalle’s invariance principle [35], if $R_{0}<1$, then $E^{0}$ is globally asymptotically stable. □

## Appendix D. The Proof of Theorem 3

**Proof****.** Construct a Lyapunov function as follows:

$$V\left(t\right)=\frac{1}{2}{\left(\left(I_{K_{i}}\left(t\right)-I_{K_{i}}^{*}\right)+\left(L_{K_{i}}\left(t\right)-L_{K_{i}}^{*}\right)+\left(S_{K_{i}}\left(t\right)-S_{K_{i}}^{*}\right)+\left(R_{K_{i}}\left(t\right)-R_{K_{i}}^{*}\right)\right)}^{2},$$

then

$$\begin{array}{cc} V^{{}^{\prime }}\left(t\right)=\; & (I_{K_{i}}\left(t\right)-I_{K_{i}}^{*}+L_{K_{i}}\left(t\right)-L_{K_{i}}^{*}+S_{K_{i}}\left(t\right)-S_{K_{i}}^{*}+R_{K_{i}}\left(t\right)-R_{K_{i}}^{*})\times \\ \; & \left(I_{K_{i}}^{{}^{\prime }}\left(t\right)+L_{K_{i}}^{{}^{\prime }}\left(t\right)+S_{K_{i}}^{{}^{\prime }}\left(t\right)+R_{K_{i}}^{{}^{\prime }}\left(t\right)\right)\\ =\; & (I_{K_{i}}\left(t\right)-I_{K_{i}}^{*}+L_{K_{i}}\left(t\right)-L_{K_{i}}^{*}+S_{K_{i}}\left(t\right)-S_{K_{i}}^{*}+R_{K_{i}}\left(t\right)-R_{K_{i}}^{*})\times \\ \; & \left(\mu -\mu I_{K_{i}}\left(t\right)-\mu L_{K_{i}}\left(t\right)-\mu S_{K_{i}}\left(t\right)-\mu R_{K_{i}}\left(t\right)\right)\\ =\; & (I_{K_{i}}\left(t\right)-I_{K_{i}}^{*}+L_{K_{i}}\left(t\right)-L_{K_{i}}^{*}+S_{K_{i}}\left(t\right)-S_{K_{i}}^{*}+R_{K_{i}}\left(t\right)-R_{K_{i}}^{*})\times \\ \; & \mu (I_{K_{i}}^{*}+L_{K_{i}}^{*}+S_{K_{i}}^{*}+R_{K_{i}}^{*}-I_{K_{i}}\left(t\right)-L_{K_{i}}\left(t\right)-S_{K_{i}}\left(t\right)-R_{K_{i}}\left(t\right))\\ =\; & -\mu {\left(I_{K_{i}}\left(t\right)-I_{K_{i}}^{*}+L_{K_{i}}\left(t\right)-L_{K_{i}}^{*}+S_{K_{i}}\left(t\right)-S_{K_{i}}^{*}+R_{K_{i}}\left(t\right)-R_{K_{i}}^{*}\right)}^{2}\\ \leq & \;0. \end{array}$$

Thus, $E^{*}$ is globally asymptotically stable if $R_{0}>1$. □

## Appendix E. The Proof of Theorem 4

**Proof****.** Let $I_{K_{i}}\left(t\right)=I_{K_{i}}^{*}\left(t\right)$, $L_{K_{i}}\left(t\right)=L_{K_{i}}^{*}\left(t\right)$, $S_{K_{i}}\left(t\right)=S_{K_{i}}^{*}\left(t\right)$, $R_{K_{i}}\left(t\right)=R_{K_{i}}^{*}\left(t\right)$, and differentiate Equation (5) with respect to $I_{K_{i}}\left(t\right)$, $L_{K_{i}}\left(t\right)$, $S_{K_{i}}\left(t\right)$, $R_{K_{i}}\left(t\right)$, one obtains

$$\begin{array}{c} \left\{\begin{array}{cc} \frac{d\lambda_{1K_{i}}\left(t\right)}{dt}= & -\frac{\partial H}{\partial I_{K_{i}}\left(t\right)}\\ = & -\{\lambda_{1K_{i}}\left(t\right)\left(-\frac{\left|\right|K_{i}\left|\right|\Theta^{*}\left(t\right)}{1+a\Theta^{*}\left(t\right)}-\mu \right)+\lambda_{2K_{i}}\left(t\right)\frac{\alpha_{1}\left|\right|K_{i}\left|\right|\Theta^{*}\left(t\right)}{1+a\Theta^{*}\left(t\right)}\\ \; & +\lambda_{3K_{i}}\left(t\right)\frac{\alpha_{2}\left|\right|K_{i}\left|\right|\Theta^{*}\left(t\right)}{1+a\Theta^{*}\left(t\right)}+\lambda_{4K_{i}}\left(t\right)\frac{\left(1-\alpha_{1}-\alpha_{2}\right)\left|\right|K_{i}\left|\right|\Theta^{*}\left(t\right)}{1+a\Theta^{*}\left(t\right)}\},\\ \frac{d\lambda_{2K_{i}}\left(t\right)}{dt}= & -\frac{\partial H}{\partial L_{K_{i}}\left(t\right)}\\ = & -\{\lambda_{2K_{i}}\left(t\right)\left(-\frac{\left|\right|K_{i}\left|\right|\Theta^{*}\left(t\right)}{1+a\Theta^{*}\left(t\right)}-\mu \right)+\lambda_{3K_{i}}\left(t\right)\frac{\beta ||K_{i}\left|\right|\Theta^{*}\left(t\right)}{1+a\Theta^{*}\left(t\right)}\\ \; & +\lambda_{4K_{i}}\left(t\right)\frac{\left(1-\beta \right)||K_{i}\left|\right|\Theta^{*}\left(t\right)}{1+a\Theta^{*}\left(t\right)}\},\\ \frac{d\lambda_{3K_{i}}\left(t\right)}{dt}= & -\frac{\partial H}{\partial S_{K_{i}}\left(t\right)}\\ = & -\{-\lambda_{1K_{i}}\left(t\right)\frac{\left|\right|K_{i}\left|\right|I_{K_{i}}^{*}\left(t\right)\Psi \left(i\right)}{{\left(1+a\Theta^{*}\left(t\right)\right)}^{2}}+\lambda_{2K_{i}}\left(t\right)(\frac{\alpha_{1}\left|\right|K_{i}\left|\right|I_{K_{i}}^{*}\left(t\right)\Psi \left(i\right)}{{\left(1+a\Theta^{*}\left(t\right)\right)}^{2}}\\ \; & -\frac{\left|\right|K_{i}\left|\right|L_{K_{i}}^{*}\left(t\right)\Psi \left(i\right)}{{\left(1+a\Theta^{*}\left(t\right)\right)}^{2}}+bu_{K_{i}}^{*}\left(t\right))+\lambda_{3K_{i}}\left(t\right)(\frac{\alpha_{2}\left|\right|K_{i}\left|\right|I_{K_{i}}^{*}\left(t\right)\Psi \left(i\right)}{{\left(1+a\Theta^{*}\left(t\right)\right)}^{2}}\\ \; & +\frac{\beta ||K_{i}\left|\right|L_{K_{i}}^{*}\left(t\right)\Psi \left(i\right)}{{\left(1+a\Theta^{*}\left(t\right)\right)}^{2}}-\left(\gamma +\mu \right)-u_{K_{i}}^{*}\left(t\right))\\ \; & +\lambda_{4K_{i}}\left(t\right)(\frac{\left(1-\alpha_{1}-\alpha_{2}\right)\left|\right|K_{i}\left|\right|I_{K_{i}}^{*}\left(t\right)\Psi \left(i\right)}{{\left(1+a\Theta^{*}\left(t\right)\right)}^{2}}\\ \; & +\frac{\left(1-\beta \right)||K_{i}\left|\right|L_{K_{i}}^{*}\left(t\right)\Psi \left(i\right)}{{\left(1+a\Theta^{*}\left(t\right)\right)}^{2}}+\gamma +\left(1-b\right)u_{K_{i}}^{*}\left(t\right))\},\\ \frac{d\lambda_{4K_{i}}\left(t\right)}{dt}= & -\frac{\partial H}{\partial R_{K_{i}}\left(t\right)}\\ = & -\{\lambda_{1K_{i}}\left(t\right)\omega +\lambda_{4K_{i}}\left(t\right)\left(-\left(\omega +\mu \right)\right)\}. \end{array}\right. \end{array}$$

By the optimal conditions, one has

$$\frac{\partial H}{\partial u_{K_{i}}\left(t\right)}=A_{K_{i}}u_{K_{i}}^{*}\left(t\right)+b\lambda_{2K_{i}}\left(t\right)S_{K_{i}}^{*}\left(t\right)-\lambda_{3K_{i}}\left(t\right)S_{K_{i}}^{*}\left(t\right)+\left(1-b\right)\lambda_{4K_{i}}\left(t\right)S_{K_{i}}^{*}\left(t\right)=0.$$

Then, one can get

$$\begin{array}{c} u_{K_{i}}^{*}\left(t\right)=\left\{\begin{array}{cc} \; & 0,\frac{B_{K_{i}}\left(t\right)}{A_{K_{i}}}S_{K_{i}}^{*}\left(t\right)<0,\\ \; & \frac{B_{K_{i}}\left(t\right)}{A_{K_{i}}}S_{K_{i}}^{*}\left(t\right),\frac{B_{K_{i}}\left(t\right)}{A_{K_{i}}}S_{K_{i}}^{*}\left(t\right)\leq 1,\\ \; & 1,\frac{B_{K_{i}}\left(t\right)}{A_{K_{i}}}S_{K_{i}}^{*}\left(t\right)>1, \end{array}\right. \end{array}$$

where $B_{K_{i}}\left(t\right)=-b\lambda_{2K_{i}}\left(t\right)+\lambda_{3K_{i}}\left(t\right)-\left(1-b\right)\lambda_{4K_{i}}\left(t\right)$. Thus, $u_{K_{i}}^{*}\left(t\right)=\max\left\{\min\left\{\frac{\left(-\lambda_{2K_{i}}\left(t\right)b+\lambda_{3K_{i}}\left(t\right)-\left(1-b\right)\lambda_{4K_{i}}\left(t\right)\right)S_{K_{i}}^{*}\left(t\right)}{A_{K_{i}}},1\right\},0\right\},i=1,2,\cdots ,n.$□

## References

[1] Liubov B. (2021). Rumors in Human Life.

[2] Daley J., Kendall G. (1964). Epidemics and rumors. Nature.

[3] Maki D., Thompson M. (1973). Mathematical Models and Applications: With Emphasis on the Social, Life, and Management Sciences.

[4] Zanette D. (2001). Critical behavior of propagation on small-world networks. Phys. Rev. E.

[5] Moreno Y., Nekovee M., Pacheco A. (2004). Dynamics of rumor spreading in complex networks. Phys. Rev. E.

[6] Xia L., Jiang G., Song B., Song Y. (2015). Rumor spreading model considering hesitating mechanism in complex social networks. Phys. A.

[7] Zan Y. (2018). DSIR double-rumors spreading model in complex networks. Chaos Solitons Fractals.

[8] Zhu L., Yang F., Guan G., Zhang Z. (2021). Modeling the dynamics of rumor diffusion over complex networks. Inf. Sci..

[9] Yang A., Huang X., Cai X., Zhu X. (2019). ILSR rumor spreading model with degree in complex network. Phys. A Stat. Mech. Appl..

[10] Yu S., Yu Z., Jiang H., Li J. (2021). Dynamical study and event-triggered impulsive control of rumor propagation model on heterogeneous social network incorporating delay. Chaos Solitons Fractals.

[11] Li J., Jiang H., Mei X., Hu C., Zhang G. (2020). Dynamical analysis of rumor spreading model in multi-lingual environment and heterogeneous complex networks. Inf. Sci..

[12] Yang S., Jiang H., Hu C. (2020). Dynamics of the rumor-spreading model with hesitation mechanism in heterogeneous networks and bilingual environment. Adv. Differ. Equ..

[13] Tong X., Jiang H., Chen X., Yu S., Li J. (2022). Dynamic analysis and optimal control of rumor spreading model with recurrence and individual behaviors in heterogeneous networks. Entropy.

[14] Capasso V., Serio G. (1978). A generalization of the Kermack-McKendrick deterministic epidemic model. Math. Biosci..

[15] Zhang T., Teng Z. (2008). Global asymptotic stability of a delayed SEIRS epidemic model with saturation incidence. Chaos Solitons Fractals.

[16] Xu R., Ma Z. (2010). Global stability of a delayed SEIRS epidemic model with saturation incidence rate. Nonlinear Dyn..

[17] Parsamanesh M., Erfanian M. (2021). Stability and bifurcations in a discrete-time SIVS model with saturated incidence rate. Chaos Solitons Fractals.

[18] Guan G., Guo Z. (2022). Bifurcation and stability of a delayed SIS epidemic model with saturated incidence and treatment rates in heterogeneous networks. Appl. Math. Model..

[19] Chen S., Jiang H., Li L., Li J. (2020). Dynamical behaviors and optimal control of rumor propagation model with saturation incidence on heterogeneous networks. Chaos Solitons Fractals.

[20] Yue X., Huo L. (2022). Analysis of the stability and optimal control strategy for an ISCR rumor propagation model with saturated incidence and time delay on a scale-free network. Mathematics.

[21] Jiang X., Wang Z., Liu W. (2019). Information dissemination in dynamic hypernetwork. Phys. A.

[22] Grilli J., Barabás G., Michalska-Smith M., Allesina S. (2017). Higher-order interactions stabilize dynamics in competitive network models. Nature.

[23] Li W., Xue X., Pan L., Lin T., Wang W. (2022). Competing spreading dynamics in simplicial complex. Appl. Math. Comput..

[24] Schaub M., Benson A., Horn P., Lippner G., Jadbabaie A. (2020). Random walks on simplicial complexes and the normalized hodge 1-Laplacian. SIAM Rev..

[25] Iacopini I., Petri G., Barrat A., Latora V. (2019). Simplicial models of social contagion. Nat. Commun..

[26] Lucas M., Cencetti G., Battiston F. (2020). A multi-order Laplacian for synchronization in higher-order networks. Phys. Rev. Res..

[27] Gambuzza L., Patti F., Gallo L., Lepri S., Romance M., Criado R., Frasca M., Latora V., Boc-caletti S. (2021). Stability of synchronization in simplicial complexes. Nat. Commun..

[28] De-Arruda G., Petri G., Moreno Y. (2020). Social contagion models on hypergraphs. Phys. Rev. Res..

[29] Alvarez-Rodriguez U., Battiston F., Arruda G. (2021). Evolutionary dynamics of higher-order interactions in social networks. Nat. Hum. Behav..

[30] Landry N., Restrepo J. (2020). The effect of heterogeneity on hypergraph contagion models. Chaos.

[31] Zhang Z., Mei X., Jiang H., Luo X., Xia Y. (2023). Dynamical analysis of Hyper-SIR rumor spreading model. Appl. Math. Comput..

[32] Lenhart S., Workman J. (2007). Optimal Control Applied to Biological Models.

[33] Fleming W., Rishel R. (1975). Deterministic and Stochastic Optimal Control.

[34] Yu S., Yu Z., Jiang H., Yang S. (2021). The dynamics and control of 2I2SR rumor spreading models in multilingual online social networks. Inf. Sci..

[35] LaSalle J. (1976). The Stability of Dynamical Systems.

